# Making Connections: Integrative Signaling Mechanisms Coordinate DNA Break Repair in Chromatin

**DOI:** 10.3389/fgene.2021.747734

**Published:** 2021-09-29

**Authors:** Anthony Sanchez, Doohyung Lee, Dae In Kim, Kyle M. Miller

**Affiliations:** ^1^Department of Molecular Biosciences, The University of Texas at Austin, Austin, TX, United States; ^2^Institute for Cellular and Molecular Biology, The University of Texas at Austin, Austin, TX, United States; ^3^Livestrong Cancer Institutes, Dell Medical School, The University of Texas at Austin, Austin, TX, United States

**Keywords:** DNA damage, chromatin, R-loops, DNA repair, protein domains, nucleic acids, genome integrity

## Abstract

DNA double-strand breaks (DSBs) are hazardous to genome integrity and can promote mutations and disease if not handled correctly. Cells respond to these dangers by engaging DNA damage response (DDR) pathways that are able to identify DNA breaks within chromatin leading ultimately to their repair. The recognition and repair of DSBs by the DDR is largely dependent on the ability of DNA damage sensing factors to bind to and interact with nucleic acids, nucleosomes and their modified forms to target these activities to the break site. These contacts orientate and localize factors to lesions within chromatin, allowing signaling and faithful repair of the break to occur. Coordinating these events requires the integration of several signaling and binding events. Studies are revealing an enormously complex array of interactions that contribute to DNA lesion recognition and repair including binding events on DNA, as well as RNA, RNA:DNA hybrids, nucleosomes, histone and non-histone protein post-translational modifications and protein-protein interactions. Here we examine several DDR pathways that highlight and provide prime examples of these emerging concepts. A combination of approaches including genetic, cellular, and structural biology have begun to reveal new insights into the molecular interactions that govern the DDR within chromatin. While many questions remain, a clearer picture has started to emerge for how DNA-templated processes including transcription, replication and DSB repair are coordinated. Multivalent interactions with several biomolecules serve as key signals to recruit and orientate proteins at DNA lesions, which is essential to integrate signaling events and coordinate the DDR within the milieu of the nucleus where competing genome functions take place. Genome architecture, chromatin structure and phase separation have emerged as additional vital regulatory mechanisms that also influence genome integrity pathways including DSB repair. Collectively, recent advancements in the field have not only provided a deeper understanding of these fundamental processes that maintain genome integrity and cellular homeostasis but have also started to identify new strategies to target deficiencies in these pathways that are prevalent in human diseases including cancer.

## Introduction

**FIGURE 1:**
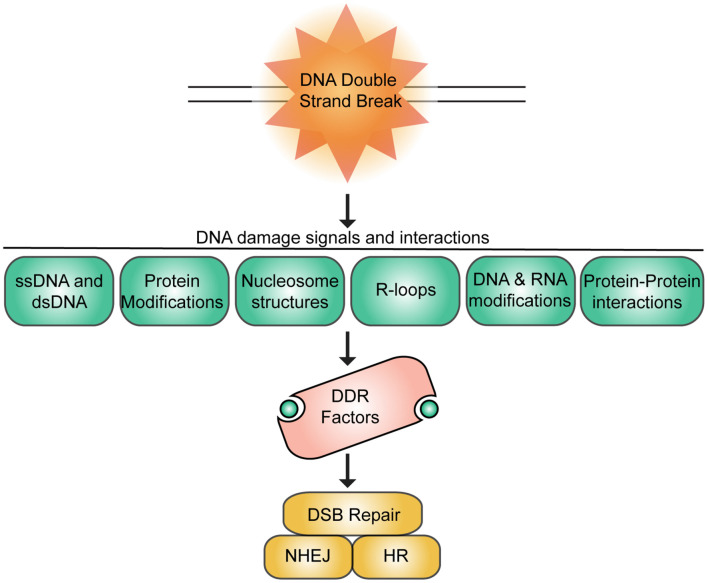
Summary of DNA damage signals and interactions at DNA double-strand breaks. DSBs signal various chromatin associated signals that promote interactions and recruitment of DNA repair factors to breaks to engage DSB repair primarily by homologous recombination repair (HR) or non-homologous end-joining (NHEJ).

DNA lesions trigger the rapid mobilization of numerous DNA damage response (DDR) proteins to the damage site where they function to not only repair the break but to also coordinate other DDR activities with additional ongoing cellular functions including transcription, replication, chromatin organization, cell cycle progression, and proliferation. Considering the vast number of proteins that assemble at breaks and the various DDR activities that they regulate, the importance of coordinating these interactions both physically and kinetically is clear. Cells use multivalent binding interactions with diverse biomolecules at the DNA lesion, whose environment is within chromatin, to control molecular signals that promote detection, processing, and repair of breaks (Figure 1). The various biomolecules that can be encountered at breaks include unmodified and modified nucleic acids of various structures (e.g., ssDNA and dsDNA, RNA and DNA, DNA and RNA methylation), nucleosomes (e.g., acidic patch region, core and variant histones), histone and protein modifications, as well as other DDR and chromatin factors (Ciccia and Elledge, 2010; Leung et al., 2014; Agarwal and Miller, 2016; Kim et al., 2019; Lanz et al., 2019; Bader et al., 2020; Skrajna et al., 2020; Sriraman et al., 2020; Tan and Huen, 2020; Fijen and Rothenberg, 2021; Klaric et al., 2021; Lee et al., 2021; Par et al., 2021). The nucleosome, which contains 147 bp of DNA wrapped around two copies of four core histones, constitutes a repetitive structure within cells that organizes the genome, while also playing an essential role in the processing of breaks. Core histone proteins that make up the nucleosome are highly modified by numerous post-translational modifications (PTM), including phosphorylation, methylation, ubiquitination, and acetylation. PTMs on histones regulate chromatin structure and function, playing essential roles in DNA-based processes including DNA repair (Thompson et al., 2013; Bowman and Poirier, 2015; Kim et al., 2019). Modified histones create a highly heterogeneous habitat in which damage can occur across the genome. Upon DNA damage, additional signaling events result in a cascade of PTM alterations within chromatin and associated repair proteins that attract DNA damage response factors to the break, where additional signaling events take place to create a repair-competent environment often at the expense of processes that were occurring pre-DNA damage. A prime example of this is transcription, which is repressed proximal to break sites to reduce conflicts between these pathways (Caron et al., 2019; Puget et al., 2019; Tan and Huen, 2020; Ui et al., 2020). Chromatin and DDR proteins contain diverse functional domains capable of interacting with the various signals that are found at the DNA break site. It is through the engagement of these multiple interactions that proteins recognize breaks within chromatin and mount a DNA damage response, which involves the transmission of signals both on chromatin and through the cell that ultimately coordinate DNA repair activities with other cellular actions (e.g., transcription, replication, and cell cycle progression). Some examples of the types of proteins that participate in the DDR include histone modifying enzymes, histone chaperones, chromatin remodelers, DNA and RNA binding and modifying enzymes, as well as DNA repair proteins themselves (Ciccia and Elledge, 2010; Polo and Jackson, 2011; Price and D’Andrea, 2013; Chiu et al., 2017; Klaric et al., 2021; Par et al., 2021). Thus, at sites of DNA lesions, these diverse sets of factors must interact with many different biomolecules to coordinate their response both in space and in time to initiate, promote and conclude DNA damage signaling and repair activities. Given the complex nature of these interactions, it is not surprising that defects in these pathways result in genome instability, a known contributor to human diseases including cancer, neurodegeneration, immunodeficiency and aging (Jackson and Bartek, 2009; McKinnon, 2017; Schumacher et al., 2021).

DNA double-strand breaks (DSBs), pose a serious threat to genomic integrity and therefore need to be repaired in an efficient and timely fashion. The repair of DSBs in mammalian cells typically proceeds through one of two main pathways, homologous recombination (HR) or non-homologous end-joining (NHEJ) (Scully et al., 2019). Repair of DSBs by homologous recombination is most-prevalent in S and G2 cell cycle phases due to the fact that this repair pathway is templated and uses a homologous sequence (i.e., sister chromatid) to complete error-free repair. The primary initiating event for HR repair is the recruitment of CtIP and the MRN complex to the DSB site where these factors generate a 3′ DNA overhang using endo-and exo-nuclease activities (Lamarche et al., 2010; Makharashvili and Paull, 2015; Keijzers et al., 2016). The 3′ overhang functions to both inhibit engagement of the NHEJ pathway and to also initiate additional activities that promote HR repair. In addition to HR, several other repair pathways can act on resected ends to promote their repair. These pathways include alternative end-joining and single-strand annealing. These pathways are less frequently engaged in HR-proficient cells but appear to play important functions in cells where HR is impaired. We refer readers to several recent reviews on this topic (Ceccaldi et al., 2016; Verma and Greenberg, 2016; Scully et al., 2019; Zhang and Gong, 2021). In HR, the single stranded DNA overhang is initially bound by RPA but is later replaced by the recombinase RAD51 through a pathway dependent on BRCA1, PALB2 and BRCA2 (Prakash et al., 2015; Kawale and Sung, 2020). RAD51 facilitates the invasion of the 3′ overhang into the homologues template *via* D-loop formation, where synthesis dependent repair occurs (Sung et al., 2003). DNA end resection is tightly regulated by various mechanisms including cell cycle and DDR factors. CDK-mediated end resection and HR regulation involves the phosphorylation of CtIP on Thr847 (Huertas et al., 2008; Huertas and Jackson, 2009). Given that CtIP interacts with and assists in promoting MRE11 function (Sartori et al., 2007; Anand et al., 2016), CtIP acts as a sensor for the cell cycle, as a CDK substrate, and transmits the information to start resection. BRCA1 also regulates DNA end resection, including through its ability to interact with CtIP in a phospho-specific manner (Yu and Chen, 2004; Yu et al., 2006; Chen et al., 2008; Yun and Hiom, 2009). Mutations of either BRCA1 or BRCA2 increase cancer risk in several different tumor types, including breast and ovarian, highlighting the importance of DSB repair factors in maintaining genome integrity and suppressing human diseases such as cancer (Li and Greenberg, 2012; Lord and Ashworth, 2016).

Unlike HR, NHEJ is a non-templated DSB repair pathway that engages the broken DNA ends and ligates them back together with little to no DNA end resection. Upon DSB formation, DNA ends are first recognized and protected from digestion by the KU70-KU80 complex (Doherty and Jackson, 2001; Chang et al., 2017). Depending on the physical features of the DNA ends, various additional NHEJ proteins are recruited including the kinase DNA-PKcs. Some breaks are re-ligated together with no end processing if the DNA ends are blunt and compatible. If incompatible ends are present, DNA-PKcs works with various other NHEJ proteins including the nuclease Artemis and polymerases to process the ends before ligation. The XRCC4-LIG4 complex is then recruited to the break to catalyze the re-ligation of the two broken DNA ends. PAralog of XRCC4 and XLF, (PAXX) interacts with KU70-KU80 to stabilize these complexes on damaged DNA to promote NHEJ in a manner independent of any apparent DNA binding activities (Ochi et al., 2015). Since some processing can occur to prepare the ends for joining and no template is used, some genetic material can be deleted or added into the break site (Rodgers and McVey, 2016). These properties of NHEJ make this DSB repair pathway more mutagenic and error-prone compared to HR.

An important question to consider is how DSB repair pathway choice is determined? Given that NHEJ is non-templated, this repair pathway occurs throughout all cell cycle phases and is believed to be the prominent repair pathway in mammalian cells (Chang et al., 2017). For HR, the resection machinery is active during S/G2 when a sister-chromatid template is present. However, in S/G2, it has been calculated that NHEJ is still the more actively engaged pathway compared to HR (Beucher et al., 2009). In addition to the cell cycle phase, other factors have also been proposed to regulate DSB pathway choice including transcription, replication, and chromatin modifications (Shrivastav et al., 2008; Marnef et al., 2017; Scully et al., 2019). The engagement of multivalent interactions also influences the pathways utilized to repair DSBs. For example, while the antagonistic relationship between the non-homologous end-joining promoting factor 53BP1 and the homologous recombination protein BRCA1 is well established, these factors utilize multiple signal recognition mechanisms within chromatin at damage sites to determine DSB repair pathway choice throughout the cell cycle (see below).

The integration of multiple interactions controls other non-DNA repair factors that influence DNA repair through their regulation of chromatin-related functions. Several factors, including the Polycomb repressive complex 1 and 2 (PRC1/2) and the nucleosome remodeling and deacetylase (NuRD) complex, can function in gene regulation through their ability to bind and alter chromatin structure and function (Lai and Wade, 2011; Basta and Rauchman, 2015; Yu et al., 2019; Piunti and Shilatifard, 2021), including in DNA break-induced transcriptional responses (reviewed here). Interactions of these complexes with breaks not only act at the level of regulating protein recruitment and activities but can also alter the biophysical properties of protein condensates themselves to create liquid-liquid phase separated compartments that have been shown to be important in both transcription and the DDR (Jiang et al., 2020; Peng et al., 2020; Fijen and Rothenberg, 2021). The chromatin environment proximal to DSBs can also contain diverse nucleic acid structures which can serve as an interface for DDR factors; in particular, RNA:DNA hybrids (R-loops) have recently emerged as a source and consequence of DSBs (Crossley et al., 2019; Marnef and Legube, 2021). R-loops, DNA and RNA, as well as chemically modified nucleotides, have all been shown to serve to further coordinate the recruitment and function of factors within the DDR (Allison and Wang, 2019; Bader et al., 2020; Sriraman et al., 2020; Klaric et al., 2021; Lee et al., 2021; Par et al., 2021). Here we highlight several principal examples, illustrating how multifaceted interactions within proteins and protein complexes collaborate at DNA damage sites to coordinate the DDR and DSB repair within the chromatin environment through the engagement of diverse molecular signals.

## Double-Strand Break Repair Pathway Choice Factor 53BP1

**FIGURE 2:**
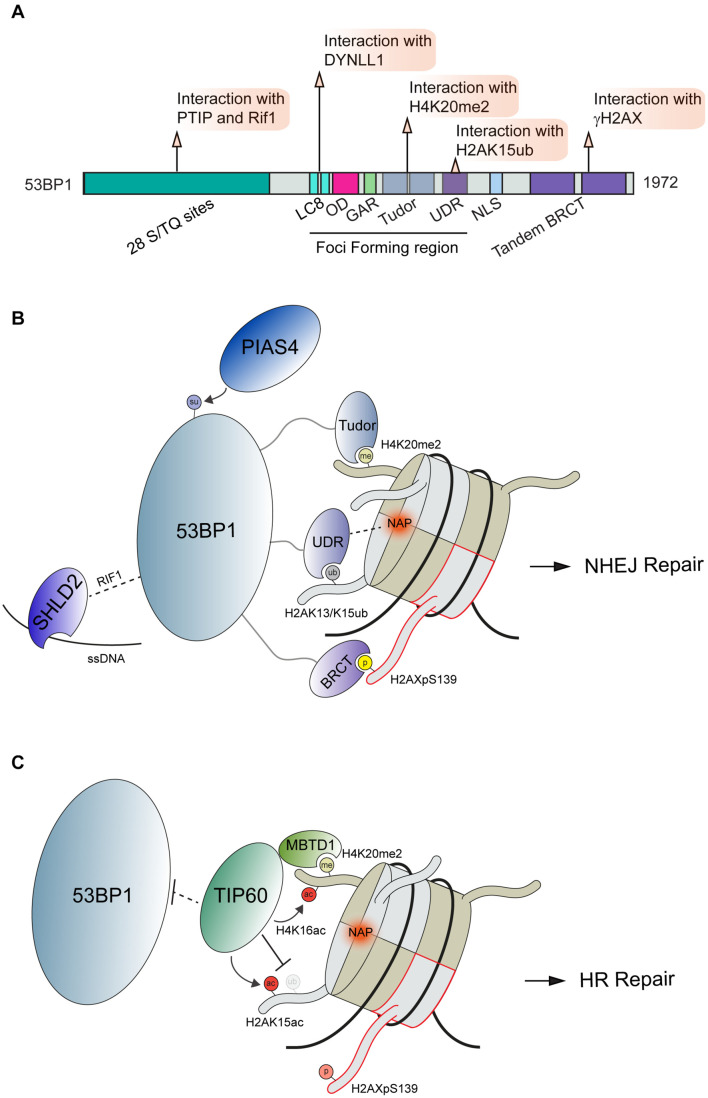
Interactions and regulation of 53BP1 during DSB repair. **(A)** Domain map of 53BP1 and DDR related interactions. **(B)** Schematic interactions between 53BP1 and chromatin proximal to DNA damage sites. 53BP1 engages with chromatin through recognition of H4K20me2 by its tandem tudor domains, interacting with the NAP and H2AK13/K15ub *via* its UDR domain and binding to H2AXpS139 *via* its BRCT domain. These interactions along with the others shown position 53BP1 on nucleosomes. **(C)** Negative regulation of 53BP1 by the histone acetyltransferase TIP60. To antagonize 53BP1 function TIP60 prevents access to H4K20me through its association with MBTD1 while also acetylating H4K16 and H2AK15. PTIP, Pax Transactivation domain-Interacting Protein; RIF1, Replication Timing Regulatory Factor 1; DYNLL1, Dynein Light Chain LC8-Type 1; OD, Oligomerization Domain; GAR, Glycine/arginine Rich Domain; UDR, Ubiquitin-Dependent Recruitment Domain; NLS, Nuclear Localization Sequence; BRCT, BRCA1 C-terminus domain; PIAS4, Protein Inhibitor Of Activated STAT 4; SHLD2, SHieLDin complex subunit 2; TIP60, 60 kDa Tat-Interactive Protein; MBTD1, MBT Domain Containing 1; NAP, Nucleosome Acidic Patch.

53BP1 is a large, 1,972 amino acid protein that contains multiple domains capable of interactions with chromatin marks and diverse DSB effector molecules at DSBs [Figure 2A; reviewed in Panier and Boulton (2014) and Mirman and de Lange (2020)]. 53BP1 engages DSB sites *via* multivalent interactions where it acts to control DSB repair pathway choice. Several specific domains are contained within the 53BP1 minimal foci forming region (FFR) that function to localize 53BP1 to DNA lesions. The FFR region of 53BP1 is composed of the dynein light chain (LC8) binding domain, oligomerization domain (OD), a glycine/arginine-rich (GAR) domain, a tandem Tudor domain, and a ubiquitin-dependent recruitment (UDR) motif (Figure 2A). The function of the GAR domain found within this region of 53BP1 remains unclear.

Upon DNA damage, 53BP1 is translocated to damaged chromatin through multiple interactions with modified histones and the nucleosome. The localization of 53BP1 to DSBs is regulated by ATM mediated phosphorylation of the histone variant H2AX on Ser139; a modification that is read by twin BRCT domains within MDC1 which in turn promotes the accumulation of the ubiquitin E3 ligases RNF8 and RNF168 (Stucki et al., 2005; Kolas et al., 2007; Mailand et al., 2007; Wang and Elledge, 2007; Doil et al., 2009; Stewart et al., 2009; Bekker-Jensen and Mailand, 2010). The DDR is driven by many such phospho-binding events that are mediated by a host of phospho-epitope binding domains including BRCT, FHA, WD40 and others [reviewed extensively in Reinhardt and Yaffe (2013)]. RNF8 ubiquitinates linker histone H1 with K63-linked ubiquitin chains at DNA damage sites, which are bound by ubiquitin binding motifs (UIM and MIU) within RNF168 to localize this E3 ubiquitin ligase to DNA lesions (Thorslund et al., 2015). While RNF168 contains several defined ubiquitin binding domains (Doil et al., 2009; Pinato et al., 2009; Stewart et al., 2009), many other DDR factors contain ubiquitin binding domains that bind to ubiquitin signals involved in signaling and repair DNA breaks [reviewed in Hofmann (2009) and Schwertman et al. (2016)]. The sequential recruitment of RNF8 followed by RNF168 has also been shown to involve L3MBTL2 through its recruitment by MDC1 and ubiquitination by RNF8, which serves to promote RNF168 accrual at DSBs (Nowsheen et al., 2018). Once localized to DSBs, these DDR factors regulate 53BP1 accumulation at damage sites in several ways. RNF8 and RNF168 mediated K48 poly-ubiquitin chains are placed onto L3MBTL1 and JMJD2D leading to their proteasome-mediated degradation. In undamaged conditions, L3MBTL1 and JMJD2A occupy H4K20me2 sites and prevent the recognition of this modification by 53BP1 (Acs et al., 2011; Mallette et al., 2012). At the same time, RNF168 catalyzes the mono-ubiquitination of H2A at lysine 15 (H2AK15ub) (Pinato et al., 2009; Stewart et al., 2009; Gatti et al., 2012; Mattiroli et al., 2012). This modification is recognized by the ubiquitin-dependent recruitment (UDR) domain of 53BP1 (Fradet-Turcotte et al., 2013). 53BP1 is retained at DSB through an additional recognition of H4K20me2, which is mediated by the tandem Tudor domains (Botuyan et al., 2006). These interactions between 53BP1 and modified histones have been further characterized using Cryo-EM (Wilson et al., 2016). Using H4K20me2 and H2AK15ub modified nucleosomes, this work revealed the structural details of 53BP1 bivalent interactions with these histone marks as well as identified an additional interaction surface between the nucleosome acidic patch and the 53BP1 UDR domain. The nucleosome acidic patch has emerged as a vital interaction hub on the nucleosome for many DDR factors in addition to 53BP1, including RNF168 (Leung et al., 2014; Mattiroli et al., 2014; Agarwal and Miller, 2016). In the case of 53BP1 and H2AK15ub recognition, it was found to be reliant on the presence of two arginine fingers in H2A and the 53BP1 UDR domain association with the nucleosome acidic patch [Figure 2B; (Wilson et al., 2016)]. Thus, these studies reveal the complex nature of 53BP1 regulation at break sites within chromatin, which utilizes multiple interactions to govern its recruitment and activities at breaks.

Recruitment and retention of 53BP1 by two different chromatin modifications likely provides a mechanism to ensure 53BP1 specifically associates with DNA damage sites by using a combination of signals that alone are not sufficient for binding but together tether 53BP1 to the break to elicit its response. In addition to the competing mechanisms with L3MBTL1 and JMJD2A (Min et al., 2007; Lee et al., 2008), 53BP1 is also regulated by histone acetylation. In response to DNA damage, the histone acetyltransferase TIP60 acetylates H2AK15 (H2AK15ac), which antagonizes RNF168-driven mono-ubiquitination (H2AK15ub) of the same site, a mark required for 53BP1 recruitment (Jacquet et al., 2016; Figure 2B). In this way, the mutually exclusive ubiquitination and acetylation on H2AK15 establishes a 53BP1 recruitment switching mechanism. TIP60 also acetylates H4K16, which is in close proximity to H4K20 (Tang et al., 2013). This acetylation sterically hinders the recognition of histone methylation on H4K20 by the Tudor domains of 53BP1 (Figure 2C). Thus, TIP60 acetylation on histones antagonizes both histone modification recruitment mechanisms for 53BP1, allowing for a robust attenuation of 53BP1 mediated repair by TIP60. The recruitment of 53BP1 to DNA damages sites is also controlled by the SUMOylation activity of PIAS4 as the expression and activity of this E3 SUMO ligase has been established as a requirement for 53BP1 recruitment to DSBs (Galanty et al., 2009). Interestingly, SUMOylation by PIAS4 was also found to be required for the DSB recruitment of RNF168; raising the possibility that 53BP1 regulation by PIAS4 occurs at the level of RNF168. Although 53BP1 has been found to be SUMOylated, the direct effect of this modification on 53BP1 functions has yet to be fully elucidated (Garvin and Morris, 2017; Figure 2B). We note that histones, including H2AX, are SUMOylated by PIAS4 (Chen et al., 2013), so the potential for SUMOylation to regulate 53BP1 on chromatin is also possible yet unexplored. There are likely additional mechanisms whereby PTMs regulate 53BP1 function on chromatin in the DDR.

In addition to histone methylation and ubiquitination, 53BP1 also directly interacts with γH2AX *via* its C-terminal BRCT repeat domain (Kleiner et al., 2015). Although this interaction is dispensable for 53BP1 accumulation at DNA lesions, the BRCT domain is crucial for the repair of DSBs in heterochromatin (Lee et al., 2010; Noon et al., 2010). In addition, ATM also directly phosphorylates the N-terminus of 53BP1 to allow recruitment of the effector protein Rif1, which acts in the 53BP1-Rif1-Rev7 axis to limit 5′ end resection and BRCA1 accumulation at DSB sites to facilitate NHEJ repair (Chapman et al., 2013; Di Virgilio et al., 2013; Escribano-Diaz et al., 2013; Zimmermann et al., 2013; Boersma et al., 2015; Xu et al., 2015). Phosphorylation of the 53BP1 N-terminus also serves to recruit PTIP through interactions between the PTIP BRCT domains and p-Ser25 on 53BP1 (Munoz et al., 2007; Callen et al., 2013). Once associated with 53BP1, PTIP promotes NHEJ and inhibits BRCA1 mediated HR repair through a mechanism that is still under investigation (Li and Greenberg, 2012; Callen et al., 2013; Escribano-Diaz and Durocher, 2013). In 2018, numerous labs converged on the identification of the Shieldin complex, a 3 protein complex consisting of SHLD1 (C20orf196, RINN3), SHLD2 (FAM35A,RINN2) and SHLD3 (CTC-534A2.2, RINN1) that forms a stable complex with REV7 [Dev et al., 2018; Findlay et al., 2018; Gao et al., 2018; Ghezraoui et al., 2018; Gupta et al., 2018; Noordermeer et al., 2018; Tomida et al., 2018); reviewed in Setiaputra and Durocher (2019)]. FAM35A (SHLD2), a component of the Shieldin complex, was reported to act downstream of 53BP1 (Dev et al., 2018; Gupta et al., 2018; Mirman et al., 2018). The association of 53BP1 with Shieldin is regulated by phosphorylation of the 53BP1 N-terminal region containing S/TQ repeats. Phosphorylated 53BP1 associates with the effector proteins Rif1, Shieldin, and PTIP (Munoz et al., 2007; Chapman et al., 2013). Functioning in concert with 53BP1, the Shieldin complex counteracts DNA end resection to support NHEJ. This is believed to occur in part through the ability of 53BP1-Shieldin complex to recruit CTC1-STN1-TEN1 (CST) to DSBs that together with Polα-primase act to counteract end resection by filling in resected DSBs (Mirman et al., 2018). In addition to the interaction with the 53BP1-Rif1 complex, the Shieldin complex can also bind to ssDNA *via* the SHLD2 oligonucleotide/oligosaccharide binding fold domain (Dev et al., 2018; Noordermeer et al., 2018). The ssDNA binding activity is believed to play a crucial role in tethering this complex to DNA repair intermediates to recruit the 53BP1-Rif1-Shieldin pathway to inhibit HR and promote NHEJ. In addition to the histone modifications described above, protein-protein interactions also impact 53BP1 recruitment to DSB sites. The self-dimerization of 53BP1 occurs through its OD domain independently of DNA damage; however, this domain is reported to stimulate 53BP1 accumulation at DSBs (Ward et al., 2006; Zgheib et al., 2009). An interaction between 53BP1 and dynein light chain (DYNLL1) *via* its LC8 binding domain has been reported, with this interaction promoting the retention of 53BP1 at damaged chromatin [Figure 2A; (Becker et al., 2018; West et al., 2019)]. DYNLL1 also interacts directly with MRE11 to limit its resection activity (He et al., 2018), which provides another example of how multiple protein interactions impinge on a pathway, which in this case acts to limit DNA end resection.

These studies highlight how 53BP1 promotes DNA repair as a consequence of multivalent interactions with chromatin and other proteins. The interactions with nucleosomes along with 53BP1 self-dimerization have recently been identified as mediators of 53BP1 phase separation (Kilic et al., 2019; Piccinno et al., 2019). 53BP1 nuclear bodies were found to exhibit hallmarks of liquid-like behavior when localized to DSBs. Of note, it was found that the protein AHNAK interacts with 53BP1 in its oligomerization domain, thereby regulating multimerization and phase separation (Ghodke et al., 2021). In AHNAK deficient cells, 53BP1 displays augmented phase separation that alters cellular responses to DNA damage. It has been demonstrated that several upstream DDR factors, including MDC1 and γH2AX, do not exhibit liquid-like behavior (Kilic et al., 2019). This raises the question of how the molecular interactions governing the association and dissociation of DDR factors regulate liquid condensates. One could envision that defects in this pathway could result in aberrant repair signaling and reactions resulting in mutations or inappropriate function of these protein complexes that must be tightly regulated to channel their activities to the correct genome location at the appropriate time. It is worth speculating that additional interactions among 53BP1, including proteins and other biomolecules, are likely to regulate and drive these interactions that are essential for recognizing and repairing breaks within chromatin.

## Regulation of BRCA1 by Chromatin Interactions

**FIGURE 3:**
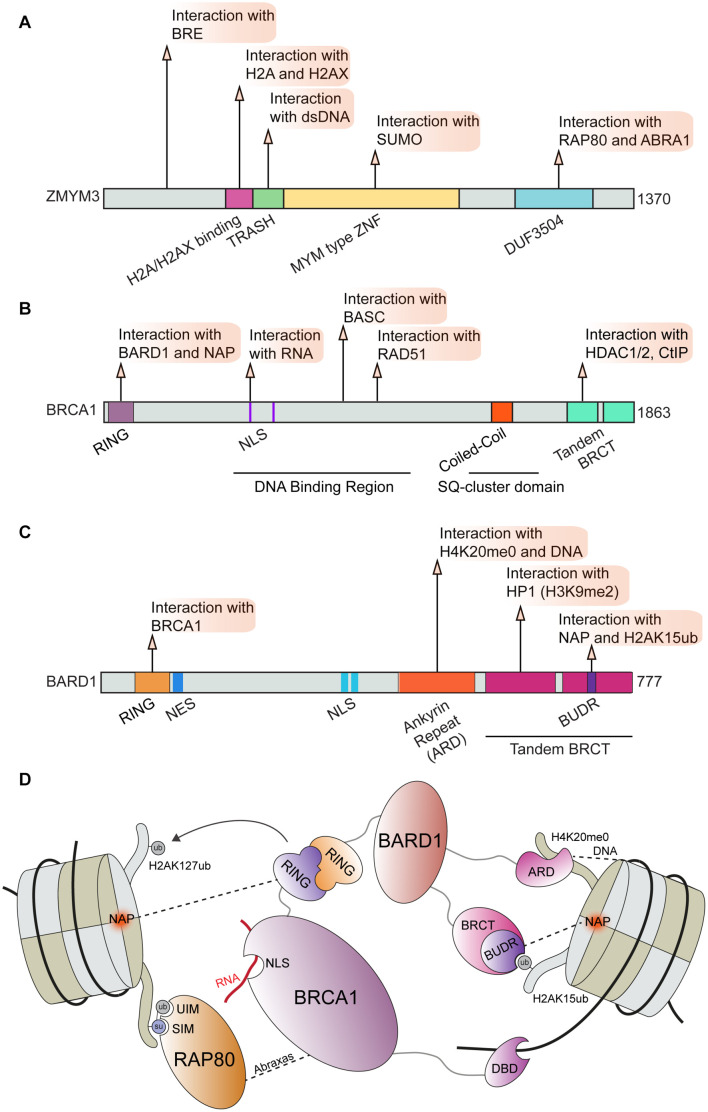
BRCA1 and associated factors in DNA repair. Domain map and DDR interactions of **(A)** ZMYM3; **(B)** BRCA1; and **(C)** BARD1. **(D)** Schematic of interactions between the BRCA1-BARD1 complex and chromatin that facilitate BRCA1 mediated DNA repair. BRCA1 can directly bind to RNA *via* its NLS region and to DNA *via* its DBD. BRCA1 binding to BARD1 through its RING domain and association with RAP80 through direct interactions with Abraxas are essential for BRCA1 function during DDR. BARD1 and RAP80 interact directly with several chromatin marks and serve to correctly position this complex at damage sites. BRE, Brain and Reproductive Organ-Expressed; RAP80, Receptor-Associated Protein 80; ABRA1, Abraxas; TRASH, Trafficking, Resistance, And Sensing Heavy Metals Domain; MYM, MYeloproliferative and Mental Retardation; DUF3504, Domain of Unknown Function; BASC, BRCA1-associated genome surveillance complex; HDAC1/2, Histone Deacetylase 1/2; CtIP, CtBP-interacting protein; RING, Really Interesting New Gene; NLS, Nuclear Localization Sequence; BRCT, BRCA1 C-terminus domain; NES, Nuclear Export Sequence; HP1, Heterochromatin Protein 1; BUDR, BARD1 ubiquitin (Ub)-dependent recruitment and BRCT-associated ubiquitin-dependent recruitment; NAP, Nucleosome Acidic Patch; UIM, Ubiquitin Interacting Motif; SIM, SUMO Interacting Motif; DBD, DNA Binding Domain.

The well-established DNA repair factor BRCA1 is known to form several distinct complexes including BRCA1-A, BRCA1-B, and BRCA1-C through alternative interactions (Chen et al., 2006; Savage and Harkin, 2015). Through these binding partners, BRCA1 serves as an integration point for several essential cellular processes and DNA repair (Ciccia and Elledge, 2010; Venkitaraman, 2014; Prakash et al., 2015; Gorodetska et al., 2019). Perturbations of BRCA1 function can act as a potent driver of cancer progression and can impact therapeutic responses to chemotherapies including platinum drugs and PARP inhibitors (Farmer et al., 2005; Li and Greenberg, 2012; Venkitaraman, 2014; Lord and Ashworth, 2016, 2017; Mylavarapu et al., 2018). Here we focus on the interactions regulating BRCA1 functions in DNA repair in chromatin; in particular the BRCA1-A complex. This complex consists of BRCA1, RAP80, BRCC36 and BRCC45, MERIT 40, and Abraxas (ABRA1) and is essential for controlling DSB repair efficiency by HR (Harris and Khanna, 2011; Wang, 2012; Rabl, 2020). The zinc finger ZMYM3 is also reported to be associated with the RAP80, ABRA1, and BRE components of the BRCA1-A complex that fine-tunes BRCA1 loading at DNA lesions (Leung et al., 2017). ZMYM3 is a member of the myeloproliferative and mental retardation (MYM)-type zinc finger protein family, which share conserved repeats of MYM-type zinc finger motifs (van der Maarel et al., 1996; Popovici et al., 1998; Smedley et al., 1999). ZMYM3 is comprised of several domains including a MYM-type zinc finger, TRASH, H2A/H2AX interacting region, a BRCA1-A complex binding area and a domain of unknown function (DUF) (Figure 3A). Collectively, these domains play an important role in regulating ZMYM3 functions at damage sites as the deletion of each motif results in impaired HR repair and genome instability (Leung et al., 2017). ZMYM3 interacts with H2A and the H2A variant H2AX, as well as double-stranded DNA *via* its H2A/H2AX binding region and TRASH domain, respectively (Leung et al., 2017). Loss of ZMYM3 results in defective BRCA1 foci at damage sites and reduced HR although how these multiple interactions within chromatin drive the function of this zinc finger protein remains poorly understood.

The ZMYM3 MYM-type zinc finger motif is also required for ZMYM3 chromatin association and efficient HR repair (Leung et al., 2017). Interactions between MYM-type zinc finger motifs and SUMO have been reported (Guzzo et al., 2014; Garvin and Morris, 2017); however, the functional consequences of the ZMYM3-SUMO interactions in regulating HR remain unknown. Given that many DDR factors involved in DSB repair are SUMOylated (Garvin and Morris, 2017), ZMYM3 may interact with SUMOylated substrates to coordinate and impact HR repair. Regulation of the BRCA1-A complex by SUMO may also occur through SUMO binding by RAP80 *via* its SUMO-interacting motif (SIM) (Anamika and Spyracopoulos, 2016; Lombardi et al., 2017). Interestingly, both SUMO binding and ubiquitin binding domains are required for RAP80 localization to DSBs; this dual recognition may fine-tune BRCA1-A complex recruitment to damage sites (Hu et al., 2012). The contribution of ubiquitination by RNF8 and RNF168 to RAP80 recognition of ubiquitin-SUMO mixed-chains still requires further investigation as the dual marks recognized by RAP80 may be conjugated by RNF4, a SUMO-targeted ubiquitin ligase (STUbL) (Guzzo et al., 2012; Chang Y. C. et al., 2021). The regulation of the BRCA1-A complex by RAP80 may also occur through interactions between ZMYM3 and RAP80. ZMYM3 directly interacts with ABRA1 and RAP80 *via* its C-terminus, and also associates with BRE through an N-terminal region (Leung et al., 2017). Interactions between ZMYM3 and RAP80, as well as ABRA1, appear to be required for the DDR function of ZMYM3 as deletion of ZMYM3 C-terminus abolishes its translocation to DNA damage sites. Even though the interaction of ZMYM3 with RAP80 and ABRA is needed for ZMYM3 damage accumulation, ZMYM3 counteracts the BRCA1 suppressive regulatory activity of RAP80 and ABRA1. Indeed, RAP80 deficiency in ZMYM3 KO cells rescues HR defects, suggesting that ZMYM3 helps antagonize RAP80 and other BRCA1A complex members to modulate HR efficiency at breaks. This finding adds new layers of regulation to the previously reported roles of RAP80 as a suppressor of BRCA1 promoted HR (Coleman and Greenberg, 2011; Hu et al., 2011). Given that all these molecules are recruited at DSB sites, ZMYM3 may balance the HR prohibitory role of BRCA1-A complex members to control BRCA1 accumulation and therefore HR at breaks, likely through its ability to interact with DNA, histones and SUMO.

ZMYM3 is only one of several chromatin factors that influence the recruitment of BRCA1 to DSBs. For example, BRCA1 and its obligate binding partner BARD1 were shown to be retained at damaged DNA sites through H3K9me2, which is mediated by the interaction between the BRCT domain of BARD1 and HP1 (Wu et al., 2015). In addition, the BRCA1 and BARD1 complex was reported to be recruited to damaged DNA sites in S-phase through an interaction with unmodified histone H4 lysine 20 (H4K20me0) (Nakamura et al., 2019; Figure 3D). In this mechanism, the ankyrin repeat domain (ARD) of BARD1 recognizes H4K20me0, a result solidified by the finding that a mutation in the ankyrin repeats disabling H4K20me0 recognition leads to a failure of BRCA1 to accumulate at DSBs (Nakamura et al., 2019). As previously described, H4K20me2 is a major binding site of 53BP1 that targets its recruitment to DNA lesions (Svobodova Kovarikova et al., 2018). In turn, dilution of methylated histones, including H4K20me2, after replication facilitates BRCA1 recruitment to promote HR repair in S-phase until the balance between unmodified and methylated H4K20 is reached in which case 53BP1-dependent NHEJ can also occur (Pellegrino et al., 2017; Simonetta et al., 2018). It is possible that *de novo* methylation of H4K20me0 at breaks in S-phase could convert this mark to H4K20me2 thereby allowing 53BP1-dependent DDR processes to occur, an option that has been observed (Tuzon et al., 2014). Regardless, these observations point to the methylation status on H4K20 as an important mechanism directing DSB repair pathway choice through 53BP1 engagement.

Recent studies have also identified additional regulatory interactions of BRCA1-BARD1 through contact with the nucleosome core particle (NCP) and various histone marks. Using a combination of biochemistry and Cryo-EM structural studies, it was found that BARD1 binds to H2AK15ub, H4K20me0, DNA and the nucleosome acidic patch (Becker et al., 2021; Dai et al., 2021; Hu et al., 2021). The Cryo-EM structures of BARD1 bound to a ubiquitinated NCP also provided new insights on the established interaction between BARD1 and H4K20me0, where it was observed that several residues on the H4 tail interact with the ARD domain of BARD1 (Dai et al., 2021; Hu et al., 2021). These results are in agreement with the predicted model for the BARD1-H4K20me0 binding interface (Nakamura et al., 2019). The ARD domain of BARD1 was also observed to bind DNA, which participated in the affinity of BARD1 to the NCP (Dai et al., 2021; Hu et al., 2021). One of the structures revealed that BARD1 interacts with the nucleosome acidic patch through the BUDR motif contained within one of the twin BRCT domains of BARD1 (Figures 3C,D; Hu et al., 2021). BRCA1 was also observed to interact with the nucleosome acidic patch (Hu et al., 2021), which is consistent with previous studies (McGinty et al., 2014; Witus et al., 2021). Finally, three investigative teams reported that a BRCT domain within BARD1, termed BUDR by two independent groups [BUDR–BARD1 ubiquitin (Ub)-dependent recruitment motif (Dai et al., 2021); BUDR–BRCT-associated ubiquitin-dependent recruitment motif (Becker et al., 2021)] binds to H2AK15ub (Becker et al., 2021; Dai et al., 2021; Hu et al., 2021). This is significant as this mark is catalyzed by RNF168, which promotes BRCA1 recruitment and this mark is also recognized by 53BP1 (Doil et al., 2009; Stewart et al., 2009; Mattiroli et al., 2012; Fradet-Turcotte et al., 2013). These findings instantly furnish a mechanism by which BRCA1-BARD1 can antagonize 53BP1 chromatin binding to promote HR through an ability to bind both H2AK15ub and H4K20me0, a mark and a histone region also recognized by 53BP1. Thus, these studies demonstrate how multivalent interactions of the BRCA1-BARD1 complex, which are summarized in Figure 3, regulate the association of this complex with damaged-containing chromatin. These interactions highlight once again the concept whereby multiple low affinity interactions cooperate to target complexes to their sites of action, which in this case is chromatin where the coordination of DSB repair pathway choice and the promotion of HR by the BRCA1-BARD1 complex takes place. We speculate that these multivalent interactions may provide additional control points for dictating how DNA repair proceeds and which BRCA1 containing complexes are recruited to sites of damage in a controlled fashion (Figure 3).

BRCA1 also interacts with other proteins at damage sites to regulate its functions. For example, the involvement of the BRCA1 coiled-coil (cc) domain in mediating interactions essential for DNA repair has recently gained attention. Coiled-coil domains are comprised of bundled alpha helices, these can be positioned in parallel or anti-parallel orientations and are established mediators of protein-protein interactions (Strauss and Keller, 2008; Truebestein and Leonard, 2016; Mier et al., 2017). The BRCA1 cc domain is known to mediate its association with PALB2 through interactions with the PALB2 cc domain. The association of BRCA1 and PALB2 is essential for BRCA1 functions in HR repair as this interaction promotes the association of BRCA1 and BRCA2 (Sy et al., 2009). The complex of PALB2 with BRCA1 is inhibited in the G1 phase as PALB2 undergoes proteasome-mediated degradation in the G1 phase which further constrains DSB repair by HR to the S and G2 cell cycle phases (Orthwein et al., 2015). Interestingly, the PALB2 cc domain was recently found to be capable of mediating PALB2 homodimerization, which may regulate the efficiency of BRCA1 mediated HR repair (Song et al., 2018). The function of PALB2 independent of BRCA1 in promoting DNA repair can impact the clinical outcome for cancer patients undergoing treatment with PARP1 inhibitors as it has been recently shown that restoring the function of PALB2 in BRCA1 null cancers also devoid of 53BP1 function can overcome resistance to PARP inhibitors (Belotserkovskaya et al., 2020). BRCA1 also associates with CtIP through interactions mediated by its cc domain (Yu et al., 2006), an interaction that has been found to facilitate replication fork stability but is dispensable for HR repair in mammalian cells (Reczek et al., 2013; Przetocka et al., 2018). The cc domain of CtIP has also been shown to mediate the dimerization of CtIP. Upon dimerization, the CtIP cc domains form a compact 4-helix bundle structure which is distinct from the CtIP-BRCA1 interaction (Dubin et al., 2004). Work remains to fully characterize BRCA1 dependent and independent functions of CtIP. Given that BRCA1 interacting partners may have functions in DNA repair independent of BRCA1 containing complexes, advancing our understanding of how these binding events are regulated will provide new insight into how DNA repair is fine-tuned. In addition to regulation *via* protein-protein interactions, BRCA1 can also impose regulation of DNA repair through its E3 ubiquitin ligase activity when in complex with BARD1 (Kalb et al., 2014). The ubiquitination of H2A on lysines 127 and 129 by the BRCA1-BARD1 complex has been identified as a prerequisite for SMARCAD1 mediated chromatin remodeling, which facilitates HR repair (Densham et al., 2016). Considering this effect on chromatin structure and DNA accessibility by BRCA1 catalyzed ubiquitination, it is not unreasonable to consider that this modification may have additional roles in regulating BRCA1 effectors in HR repair. Further work is needed to fully characterize the contribution of BRCA1 interactors and modifications mediated by BRCA1 in the regulation of DNA repair. A more complete understanding of these multivalent interactions may provide new avenues for therapeutic intervention in cancer types driven by BRCA1 dysfunction (Na et al., 2014).

## Regulation of Transcription at Double-Strand Break Sites

**FIGURE 4:**
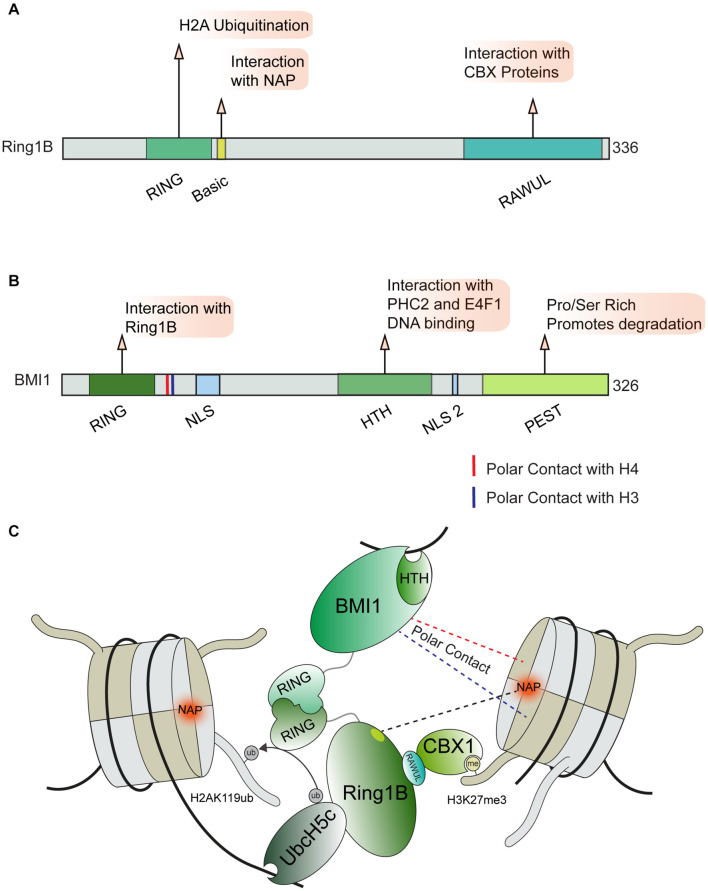
PRC1 complex and chromatin interactions involved in DNA repair. **(A)** Ring1b and **(B)** BMI1 domain maps and DDR related interactions. **(C)** Depiction of chromatin interactions exhibited by core PRC1 members BMI1 and Ring1B involved in transcriptional repression and DNA repair. BMI1 and Ring1B associate *via* their RING domains, Ring1B directly interacts with CBX1 and the E2 enzyme Ubch5c which help position the PRC1 complex for ubiquitin conjugation on H2AK119. Polar contact between BMI1 and H3 or H4 are indicated by blue and red dotted lines, respectively. BMI1, B lymphoma Mo-MLV insertion region 1 homolog; RING, Really Interesting New Gene; CBX, Chromobox Homolog; PHC2, Polyhomeotic-like protein 2; NAP, Nucleosome Acidic Patch; RAWUL, Ring-finger and WD40 associated Ubiquitin-like; NLS, Nuclear Localization Sequence; HTH, Helix Turn Helix; PEST, rich in proline (P), glutamic acid (E), serine (S), and threonine (T).

Active transcription through chromatin presents a complex physical structure containing newly synthesized RNA, the separated DNA strands, histones, and the transcription machinery (Li et al., 2007; Barnes et al., 2015; Venkatesh and Workman, 2015). This diverse environment requires regulatory factors capable of recognizing these various structures and proteins engaged at DNA lesions within sites of active transcription. This idea is exemplified by the PRC1 complex, which recognizes multiple histone marks and nucleosome features in order to regulate transcription at DSBs in addition to its roles in transcription during development (Leeb and Wutz, 2007; Aranda et al., 2015). The PRC1 complex is comprised of several subunits which can mediate distinct interactions with chromatin (Figure 4). All described variants of the PRC1 complex contain the core components Ring1B (RNF2) and a PCGF protein (most commonly BMI1) however, multiple distinct forms of PRC1 are expressed in human cells, which further contributes to the diverse interactions that this complex can accommodate (Chittock et al., 2017). The Ring1B component of PRC1 acts as an E3 ubiquitin ligase that mono-ubiquitinates histone H2A and H2AX at lysine 119 proximal to DSB sites, with this signal being associated with the repression of transcription (Tamburri et al., 2020) and promotion of the DDR (Shanbhag et al., 2010; Kim et al., 2019). However, some ubiquitination independent transcriptionally repressive functions of PRC1 have been described during normal transcriptional regulation (Pengelly et al., 2015; Tsuboi et al., 2018). Whether or not these functions also contribute to DNA damage activities of the PRC1 complex is not yet defined.

Several interactions with histones and DNA are required to correctly position the PRC1 complex so that it specifically ubiquitinates H2A or H2AX on lysine 119. The activity of Ring1B on H2A and H2AX was shown to require the nucleosome acidic patch in both biochemical and cell-based systems (Leung et al., 2014). X-ray crystallography provided structural details on how Ring1B interacts with the nucleosome; in particular, Ring1B Arg98 inserts into an H2A acidic patch by making hydrogen bonds with H2A side chain carboxylates (McGinty et al., 2014; Figures 4A,C). BMI1 participates in polar interactions with H3 and H4, however, the effect of this interaction on PRC1 activity remains undefined (Barbour et al., 2020). The positioning of the PRC1 complex on nucleosomes is further directed by interactions between the associated E2 enzyme, UBCH5C, and DNA (Bentley et al., 2011; McGinty et al., 2014). The catalytic activity of the PRC1 complex is enhanced by the contact between UBCH5C and the DNA. The multivariant binding exhibited by PRC1 may serve to promote specific functions or recruit specific PRC1 variant complexes to chromatin. We note that PRC1 has been shown to be positioned on chromatin in proximity to areas of active replication, which raises the possibility of additional interactions between PRC1 and the replisome or aberrant nucleic acid structures (e.g., R-loops), which warrants further investigations. While this localization could be attributed to known PRC1 interactions, recent reports have identified PRC1 as essential for the progression of the replication fork, processing of R-Loop structures, and the integrity of common fragile sites which may indicate a more direct role in these processes (Klusmann et al., 2018; Sanchez et al., 2020). In addition to the PRC1 complex, several other pathways that regulate H2AK119ub at break sites have been identified including PRC2, PBAF, ENL, and FRRUC complexes, for which we refer readers to recent in-depth reviews that have covered the extensive involvement of multiple complexes in repressing transcription at DNA breaks, including through the regulation of H2A ubiquitination (Caron et al., 2019; Puget et al., 2019; Tan and Huen, 2020).

**FIGURE 5:**
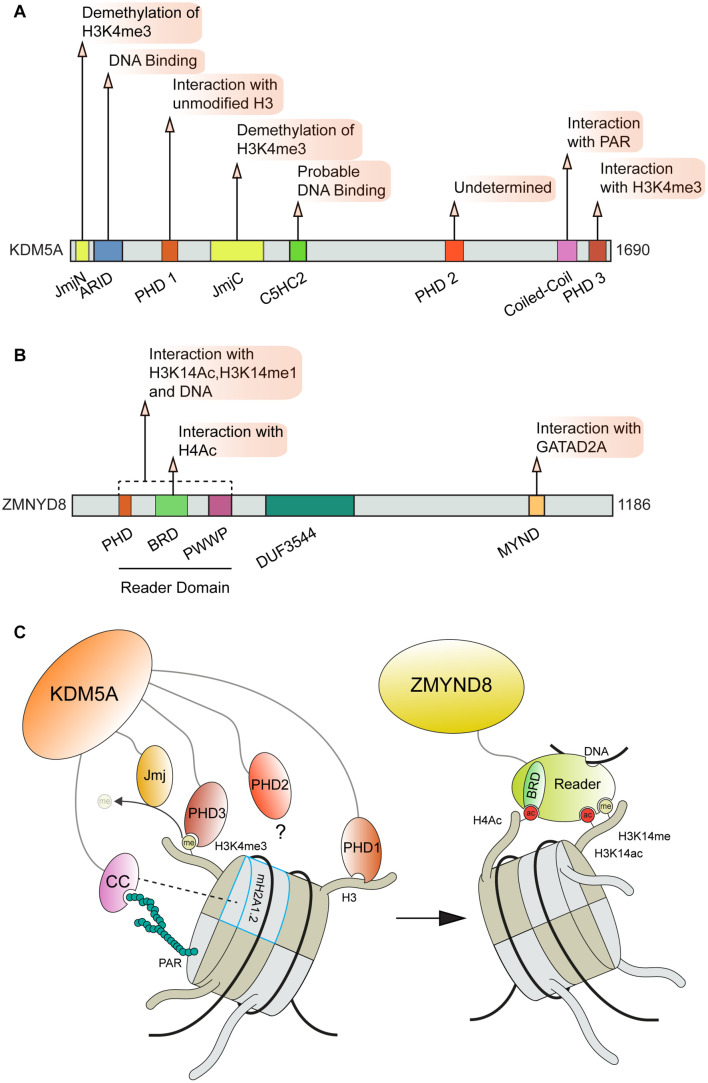
Involvement of ZMYND8 and KDM5A domains and interactions in DNA repair. **(A)** KDM5A and **(B)** ZMYN8 protein domain maps and interactions. **(C)** KDM5A interactions that facilitate its function at breaks and recruitment of ZMYND8 to damage sites. KDM5A interacts with nucleosomes through recognition of unmodified H3 tails and H3K4me3 *via* its PHD1 and PHD3 domains, respectively. KDM5A also recognizes PAR chains and macroH2A1.2 through a coiled-coil (cc) domain. ZMYND8 binding to nucleosomes is facilitated primarily through its Reader domain which recognizes H4ac, H3K14me and H3K14ac histone marks. KDM5A, Lysine-specific Demethylase 5A; Jmj, Jumonji domain; ARID, A–T Rich Interaction Domain; PHD, Plant HomeoDomain; ZMYND8, Zinc Finger MYND-Type Containing 8; BRD, Bromodomain; PWWP, “Pro-Trp-Trp-Pro” domain; DUF, Domain of Unknown Function; MYND, Myeloid, Nervy, and DEAF-1 domain; CC, Coiled Coil; PAR, Poly ADP-Ribose.

The importance of transcriptional regulation at DNA damage sites is supported by the fact that this process is controlled through multiple pathways which rely on diverse interactions with chromatin and DNA. As an *exemplum primi*, the KDM5A-ZMYND8-NuRD pathway forms multiple contacts with chromatin and modifications, which are critical for this complex to function at DNA breaks [Figure 5; (Gong et al., 2015, 2017; Savitsky et al., 2016; Spruijt et al., 2016; Gong and Miller, 2019)]. Mechanistically, KDM5A promotes transcriptional repression and DNA damage repair at DSB sites through the demethylation of histone H3 at lysine 4 (H3K4me3), which allows for the subsequent stable recruitment of ZMYND8-NuRD *via* recognition of TIP60 mediated acetylation of H4 by the ZMYND8 BRD domain (Gong et al., 2015). The association between the ZMYND8 MYND domain and the PPPLΦ domain of the NuRD complex GATAD2A subunit localizes the NuRD complex to DSB sites where it can promote DNA repair through transcriptional repression *via* nucleosome remodeling mediated by the CHD4 subunit [Figure 5B; (Gong et al., 2015; Spruijt et al., 2016)]. ZMYND8 can also engage with nucleosomes through interactions with H3K15ac and H3K14me1, which are mediated by the ZMYND8 “reader domain” (containing tandem PHD, BRD, and PWWP domains, [Figures 5B,C; (Savitsky et al., 2016)]. This domain within ZMYND8 also binds DNA (Savitsky et al., 2016). In order to support ZMYND8-NuRD recruitment to DSBs, KDM5A relies on multiple interactions on chromatin to correctly position its catalytic Jumonji C (JmjC) domain on H3. KDM5A binding to H3 is made by two plant homeodomain zinc fingers (PHD), PHD1 that recognizes the unmodified N-terminal tail of H3 (Torres et al., 2015) and PHD3 that specifically interacts with H3K4me3 (Wang et al., 2009). The interactions between KDM5A PHD1 domain and the unmodified H3 N-terminal tail also regulates KDM5A activity through induced conformational changes (Longbotham et al., 2021; Figures 5A,C). For recruitment to DNA damage sites, PHD1 but not PHD3 was required to support KDM5A translocation to breaks (Gong et al., 2017). Recently, the localization of KDM5A to sites of DNA damage was also found to be dependent on the presence of the histone variant macro H2A1.2 (mH2A1.2) and PARP1 activity (Kumbhar et al., 2021). Depletion of either mH2A1.2 or PARP1 disrupted the localization of KDM5A to DSBs and perturbed the ability of KDM5A to promote DNA repair and transcriptional repression. Interestingly, the association between KDM5A and PAR chains was found to be mediated by a previously unidentified coiled-coil domain (cc domain) within the C-terminus of KDM5A spanning residues 1,501–1,562. The presence of this domain was also found to be required to support KDM5A localization and function at break sites (Kumbhar et al., 2021). Further analysis uncovered that KDM5A exhibits preferential binding to extended PAR chains (ex. 27mer) compared to chains of shorter lengths (Figure 5C). This specificity may provide an additional layer of regulation to dictate KDM5A functions at sites of DNA damage. Importantly, cc domains have not been previously identified as a PAR binding domain (Teloni and Altmeyer, 2016) yet this domain within KDM5A binds PAR chains with an apparent affinity in the range of established PAR binding domains involved in the DDR including PBM, PBZ, and macro domains [reviewed in Teloni and Altmeyer (2016)]. This finding raises several intriguing questions about PAR and chromatin mediated interactions at DSBs. Given that approximately 10% of all proteins are predicted to contain coiled-coil domains, further explorations are warranted to characterize the role of cc domains in facilitating interactions with PAR. The role of phase separation in DNA damage response factors has gained attention recently (Pessina et al., 2019) and regions of intrinsic disorder and cc domains are known to contribute to the process of liquid-liquid phase separation (LLPS) (Anurag et al., 2012; Schuster et al., 2020). The potential for interactions between cc domains and PAR to regulate functions mediated by LLPS should be considered and may be determined by PAR binding/chain lengths and the activity of PARPs during DNA break repair.

## RNA:DNA Hybrids in Double-Strand Break Repair

R-Loops are 3-stranded RNA:DNA hybrid molecules that form when RNA transcripts hybridize with the template DNA, which poses a substantial obstacle for the replication machinery and causes genomic instability and replication associated DNA breaks (Puget et al., 2019; Brambati et al., 2020; Marnef and Legube, 2021). Structurally, R-loops consist of a region of base-paired RNA:DNA, a displaced single strand of DNA and RNA overhangs (both 3′ and 5′); these distinct nucleic acid structures can be bound by a growing number of factors to catalyze their resolution (Cristini et al., 2018; Allison and Wang, 2019). The structure of R-loops can also directly promote mutagenesis as it has been proposed that the exposed ssDNA strand is vulnerable to nucleases or DNA damaging agents (Huertas and Aguilera, 2003; Makharashvili et al., 2018). The role of RNA nucleases and helicases, including RNaseH1/2 and Senataxin, respectively, in resolving R-loop structures is now well described (Fedoroff et al., 1993; Cerritelli and Crouch, 2009; Hatchi et al., 2015; Groh et al., 2017; Parajuli et al., 2017; Cohen et al., 2018; Lockhart et al., 2019). DSB repair factors also have also been shown to directly bind to R-loops. For example, BRCA1 and the BRCA1/BARD1 complex was shown to preferentially bind R-loops over dsDNA *in vitro* and BRCA1 colocalized with R-loops in IR-treated cells, which was detected using super-resolution fluorescence microscopy (D’Alessandro et al., 2018). Interestingly, this study showed that expression of RNaseH1 in IR-treated cells impaired BRCA1 recruitment to damage sites. An association between TERRA R-loops and BRCA1 was also recently described at telomeres and it was found that BRCA1 can associate directly with TERRA RNA through interactions mediated by the BRCA1 N-terminal NLS region (Vohhodina et al., 2021). Binding of RNA *via* NLS sequences has been identified in other factors including the ribonuclease Dicer, which can be attributed to the density of positive charged amino acids in these regions that can facilitate binding to the ribonucleotide backbone (LaCasse and Lefebvre, 1995). The binding of TERRA by BRCA1 results in the suppression of TERRA transcription and promotes the repair or R-loop associated DNA damage at telomeres (Vohhodina et al., 2021). The association of BRCA1 and R-loops at sites of DNA damage may also occur through NLS mediated interactions (San Martin Alonso and Noordermeer, 2021). In addition to BRCA1, BRCA2 also promotes R-loop processing, which has been shown to be regulated by the helicase DDX5 and RNaseH2 (D’Alessandro et al., 2018; Sessa et al., 2021). In the case of DDX5, BRCA2 was shown to stimulate its helicase activity (Sessa et al., 2021). Using only the N-terminal 250 amino acids of BRCA2, which was shown to encompass the DDX5-interaction region, this fragment of BRCA2 retained the ability to stimulate DDX5 unwinding of R-loops. These results suggest that BRCA2 itself does not directly bind to R-loops but rather regulates these structures through protein interaction partners that themselves can recognize and act on R-loops. PALB2, which is found in complex with BRCA1 and BRCA2, contains strand exchange activity involving its N-terminal DNA binding domain that can also bind RNA (Deveryshetty et al., 2019). Thus, all three of these HR proteins have been shown to interact with R-loops either directly or through interaction partners.

ssDNA binding proteins involved in DSB repair have also been linked to R-loops. The role of RPA in regulating R-loop formation and resolution has been of interest for some time and RPA co-localizes with RNaseH1 (Nguyen et al., 2017). It was proposed that RPA association with R-loops was through interactions between RPA and the displaced single stranded DNA (Pokhrel et al., 2019) but more recently it has been found that RPA can directly engage with R-loops and bind to RNA with moderate affinity (Mazina et al., 2020). Finally, the most downstream factor involved in HR-mediate repair of DSBs is the recombinase RAD51, which replaces RPA on ssDNA through the activities of BRCA1, PALB2, and BRCA2. Evidence in yeast has suggested that in addition to DNA-DNA strand exchange, RAD51 can also promote DNA-RNA strand exchange that could be involved in R-loop biogenesis (Wahba et al., 2013) although another study obtained results showing that R-loops involved in genome instability form independently of RAD51 (Lafuente-Barquero et al., 2020). The RAD51 interacting protein RAD51AP1 generates R-loops *in vitro* and surprisingly was shown to generate a new recombination intermediate termed a DR-loop, which contains an R-loop within a D-loop. Like several other factors including PALB2, the ssRNA binding activity and R-loop forming ability were dependent on the DNA binding domain of RAD51AP1, suggesting that nucleic acid binding regions can multitask on various structures that form at breaks and during the repair process. It is worth noting that RAD51 in human cells has been reported to promote telomeric recruitment of TERRA *in trans* and formation of telomeric R-loops (Feretzaki et al., 2020) and this activity was found to promote telomere elongation in telomerase negative ALT positive cells. Taken together, these studies highlight the interplay between R-loops and genomic features including DSBs and telomeres. Given the prevalence of both DNA and RNA binding activities in several core HR proteins, it is tempting to speculate that regulatory mechanisms must exist through multiple binding events that function to orientate these complexes at the DNA lesion and ensure their engagement with the requisite structure intermediate rather it be of DNA or RNA origin. It is likely that the deployment of reconstituted biochemical and single molecule systems, structural studies and *in vivo* techniques including super resolution microscopy and Cryo-EM Tomography will be needed to address the challenging questions that remain for how these multi-protein molecular machines and complexes function within chromatin to sense, process, and repair DNA breaks.

The formation of stable R-loops in the genome can give rise to a unique situation where transcription and replication complexes are competing for occupancy of the same DNA template. A growing body of evidence supports a model where a significant source of R-Loop associated DNA damage results from transcription-replication conflicts (TRC), which ultimately can also lead to DSBs (Helmrich et al., 2011; Garcia-Muse and Aguilera, 2019; Puget et al., 2019; Sanchez et al., 2020). The use of novel reporter systems has demonstrated that in both bacterial and human systems, TRCs are most detrimental to cells when they occur in a “head-on” orientation, meaning that the replication and transcription complexes are moving toward each other on the DNA (Hamperl et al., 2017; Lang et al., 2017). R-loops induce DSBs through replication stalling and breakage, which is supported by their increased frequency in close proximity to DSBs (Marnef and Legube, 2021). Thus, DNA stress response pathways involved in DSB repair and replication involve R-loops. To understand mechanistically how R-loops and the proteins that regulate them are involved in these pathways, it will be helpful to identify the DNA and/or RNA binding factors, their modes of binding to various nucleic acid structures and the activities used to regulate R-Loops. This information can inform working models and insights into how these transactions work and are regulated in cells to maintain genome integrity. The speed at which this field is moving is rapid, with future studies expected to reveal the inner workings of how R-loops impact genome integrity through their functions in repair, replication and transcription.

Several reports have elucidated the involvement of DNA damage associated helicases in resolving R-loops. For example, the Fanconi Anemia (FA) helicase FANCM, as well as FANCD2 and FANCI, have all been implicated in R-Loop resolution (Okamoto et al., 2019). Interestingly, the association of FANCI-FANCD2 (ID2) with R-loops appears to be specific to the displaced ssDNA region or the free RNA overhangs and not the RNA:DNA hybrid region of the R-Loop (Liang et al., 2019). Binding of the ID2 dimer to R-loops was found to promote FANCD2 mono-ubiquitination by the FA core complex; however, the functional consequence of this event for R-Loop resolution remains poorly defined. The recognition of ssDNA and RNA overhangs by FANCD2 raises some exciting possibilities and areas for further exploration. Foremost, the identification of the region on FANCD2 capable of interacting with R-loop structures would provide more insight into how R-loop resolving factors may function. The recently identified DNA binding motifs within FANCD2 present one intriguing possibility for how FANCD2 may recognize structural features present within R-Loops (Niraj et al., 2017). FANCM has also been shown to resolve telomeric R-loops through its ATPase activity (Silva et al., 2019) and/or the interaction with the BLM-TOP3A-RMI complex (Lu et al., 2019). These findings further support a multifaceted role for canonical DDR factors which engage R-loops at structurally and potentially functionally diverse areas of the genome to promote genome integrity mechanisms. Additionally, in mutant cells where these pathways are defective, the contribution of unresolved R-loops to FA and genome instability, including through the production of TRCs and DSBs are not fully elucidated.

The role of nucleosome remodeling complexes in resolving R-loops has also recently been investigated. As a case in point, the INO80 complex was identified as a R-loop resolving factor. The INO80 complex has well established functions during replication and transcription, during which INO80 positions histones at transcription start sites and interacts with the transcription complexes RNAPII and PAF1 (Poli et al., 2017). During replication, INO80 is required for replication restart after fork stalling (Lee et al., 2014) and is necessary for replication fork progression through nucleosome bound DNA (Kurat et al., 2017). New findings now indicate that the effect of INO80 on replication fork progression may be in part due to its R-loop resolving functions. Defects in replication fork progression in INO80 deficient cells can be rescued by overexpression of RNaseH1, providing strong evidence for this idea (Prendergast et al., 2020). Strikingly, it was found that INO80 can locally resolve R-loops within chromatin at stalled forks using the LacO-LacR array system (Prendergast et al., 2020). Although the structure of the INO80 complex bound to nucleosomes has been determined by Cryo-EM (Ayala et al., 2018), it is not yet clear which subunit, activity or binding substrate is required for R-loop resolution. Nucleosome remodeling by the SWI/SNF complex has also been implicated in the resolution of R-Loops (Bayona-Feliu et al., 2021). It was found that depletion of the BRG1 subunit resulted in increased R-Loop associated damage and increased transcription-replication conflicts, indicating that SWI/SNF remodeling activity is required for resolving R-Loops resulting from head on collisions between the replisome and RNAPII. Interestingly, BRG1 co-localizes with FANCD2 at R-loops and co-depletion of these factors has an epistatic effect on cellular R-Loop formation; indicating that these complexes may work together to resolve R-Loops. This association between SWI/SNF and FA proteins is consistent with previous work describing a direct interaction between BRG1 and FANCA (Otsuki et al., 2001). As replication associated factors continue to be explored in the resolution of R-loops, the role of these additional interaction interfaces in regulating R-loop metabolism and the consequences for genome stability and DNA break formation will be an essential area of inquiry. As remodelers impact transcription, it cannot be ruled out that these activities are linked to both replication and DNA damage associated R-loop functions of these large molecular machines that interact with and function within chromatin.

Proteomic approaches have shed light on the factors that respond to R-loops (Cristini et al., 2018). For instance, the Gromak group identified over 450 R-loop interacting proteins by utilizing the S9.6 RNA:DNA hybrid antibody coupled with IP/MS analysis. Another study obtained complementary results using a biotin tagged *BAMBI* promoter and *DPP9* 3′UTR sequences, which are known sites of R-loop accumulation (Wang et al., 2018). Although these studies were performed in different cell types, there were 197 common R-loop interacting factors identified between them including known R-loop resolving factors, helicases, and proteins capable of interacting with RNA and DNA. In addition to recognizing R-loop structural features, factors responding to R-loops may also interact with chemically modified DNA or RNA present at the R-Loop (Al-Hadid and Yang, 2016; Lee et al., 2021). In support of this notion, a recent study utilizing DRIP-Seq found that the majority of R-loops throughout the genome contain N6-methyladenosine (m^6^A) RNA modifications (Abakir et al., 2020). Of note, this modification was found to be recognized by the m^6^A reader YTHDF2, which in turn promoted the degradation of the R-loop. The RNA strand of an R-loop may also contain 5-methylcytosine (m^5^C) modifications, which occurs in response to DNA damage and is catalyzed by the TRDMT1 RNA methyltransferase (Chen et al., 2020). The expression of TRDMT1 and the m^5^C modification were both found to aid the recruitment of HR repair factors RAD51 and RAD52, further highlighting the potential importance of this RNA modification in DNA repair. RAD52 has been found to bind R-loops to promote XPG mediated repair, which is involved in transcription-associated homologous recombination repair (Yasuhara et al., 2018). The contribution of m^5^C to RAD52 R-loop binding was assessed and interestingly, it was found that RAD52 binds to m^5^C modified R-loops with a higher affinity than unmodified hybrids (Chen et al., 2020). Further work is needed to determine the mode of binding to the m^5^C modification in RAD52 as currently the region binding this modification has not been determined. It is still a matter of debate about the function of R-loops at DSBs and their origins, including whether or not these structures promote or inhibit DNA repair [reviewed in Skourti-Stathaki and Proudfoot (2014); Crossley et al. (2019); Puget et al. (2019); and Marnef and Legube (2021)]. Regardless, to understand mechanistically how DDR proteins recognize and promote R-loop formation and/or resolution, it will be paramount to determine how these factors recognize and interact with R-loops, including at DNA lesions. The presence of DNA damage specific R-loop modifications presents yet another additional layer of complexity requiring further inquiries.

## Conclusion and Perspectives

**FIGURE 6:**
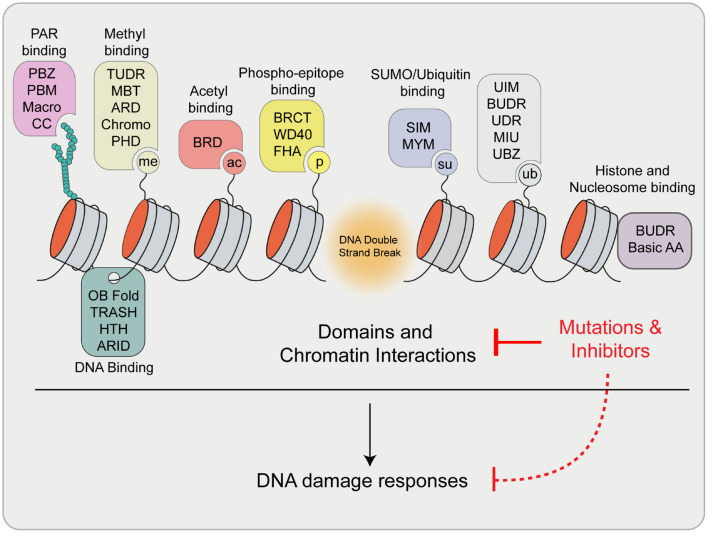
Summary of reviewed and highlighted interaction domains involved in the DDR. Upon DSB formation, many signals are generated that are recognized by specific domains within proteins involved in DNA damage signaling and repair. These proteins engage break sites and chromatin through these interactions to exert their function. Mutations and/or the use of inhibitors of these domains have the potential to disrupt these interaction and pathways, which may impact downstream DDR processes. Definitions: PBZ, PAR Binding Zinc finger; PBM, PAR Binding Motif; CC, Coiled coil; ARD, Ankyrin Repeat Domain; MBT, Malignant Brain Tumor Domain; PHD, Plant Homeodomain; BRD, Bromodomain; BRCT, BRCA1 C-terminus domain; FHA, Forkhead-associated domain; SIM, SUMO Interacting Motif; MYM, MYeloproliferative and Mental retardation; UIM, Ubiquitin Interaction Motif; BUDR, BARD1 ubiquitin (Ub)-dependent recruitment and BRCT-associated ubiquitin-dependent recruitment; UDR, Ubiquitin-Dependent Recruitment domain; MIU, Motif Interacting with Ubiquitin; UBZ, Ubiquitin Binding Zinc finger; OB Fold, Oligosaccharide-Binding Fold; TRASH, Trafficking, Resistance, And Sensing Heavy metals domain; HTH, Helix Turn Helix; ARID, A–T Rich Interaction Domain; Basic AA, Arginine Anchor.

As highlighted here, essential factors involved in DNA repair exhibit diverse binding and interactions within chromatin, which can control specific functions during the DDR and influence how DNA lesions are managed. These factors utilize a wide range of specific protein domains that are used to bind various biomolecules at sites of damage (summarized in Figure 6). The view of DNA repair is evolving and now constitutes consideration of not only protein and protein interactions and DNA binding at breaks but also the involvement of RNA structures including R-loops, modified proteins and nucleic acids, as well as other interactive signals that drive DNA repair processes. These multivariant interactions may also present potential vulnerabilities for controlling the activity of these factors. Considering the number of structurally unique protein domains required to coordinate DNA repair on chromatin, it is not surprising that many small molecule inhibitors are available that are potentially capable of disrupting the functions of these domains (Arrowsmith and Schapira, 2019; Mio et al., 2019). Chemical or peptide based inhibitors have recently been developed to target several domains that are found within DDR proteins including tandem tudor domains (Chang L. et al., 2021), PRC1 chromodomains (Stuckey et al., 2016), PHD zinc fingers (Wagner et al., 2012), bromodomains (Filippakopoulos et al., 2010) and ubiquitin interacting motifs (UIM) (Manczyk et al., 2019). In addition to their potential clinical applications, the development and availability to researchers of specific inhibitors of these repair-chromatin interactions will be advantageous for untangling and defining the specific contribution of individual contacts within these proteins and their ability to mediate DNA repair.

Altering the physical properties of chromatin bound complexes also provides a potential avenue for specifically controlling the action of repair factors. The formation of membrane-less condensates has been found to facilitate transcription (Boija et al., 2018) and promote DNA repair (Oshidari et al., 2020). With this in mind, the prospect of specifically targeting the function of specific DNA factors by inhibiting their ability to undergo phase separation emerges (Klein et al., 2020). The potential to target phase separation therapeutically also benefits from the fact that many chromatin bound factors can be found localized within distinct phase separated-complexes under different cellular conditions; an example being the multiple forms of PRC1 complexes (Chittock et al., 2017). Under specific conditions during development PRC1 can function independently of its catalytic activity and Polycomb body formation (Tsuboi et al., 2018); however, recent evidence supports enhanced functions of PRC1 through phase separation mediated by the Ph-SAM subunit of PRC1 (Seif et al., 2020). This type of movement between phase separated states may provide a method to target the localization and function of DNA repair factors at specific genomic locations. More work is needed to understand the precise regulation and function of DNA repair associated condensates and how these can be manipulated specifically without altering other biological processes that utilize these pathways. For example, transcription and repair events are intimately linked in both DSB repair and in engaging phase separation as a regulatory mechanism. It may be challenging then to uncouple one process from the other, which has always been difficult for multi-functional proteins unless separation of function mutations can be generated. The use of comprehensive CRISPR-Cas9 protein domain screens or CRISPR-dependent cytosine base editing screens that can generate protein variants can provide powerful unbiased separation of function screens to address the specific contribution of domains within proteins involved in multiple interactions and biological processes (Shi et al., 2015; Cuella-Martin et al., 2021). In addition, sequences from tumor genomes (e.g., TCGA) may provide additional insights into the function of these domains in cancer, which often exhibit defects in DDR pathways (Helleday et al., 2008; Mio et al., 2019). Determining the functional domains within DDR factors and their potential druggability and/or mutation status in cancer will likely be a valuable endeavor. This information can improve our mechanistic understanding of how DNA repair occurs on chromatin templates in cells and ultimately identify vulnerabilities and/or drug targets for therapeutic interventions in human diseases including cancer.

Given the clear connection between DNA repair and chromatin that occurs at the level of interactions between DNA repair factors and nucleic acids, nucleosomes and modified histones, the physical state of chromatin itself should be considered and may also have dramatic effects on how DNA repair proceeds (Aymard et al., 2014; Marnef et al., 2017; Fortuny and Polo, 2018; Fortuny et al., 2021). The compaction or decompaction of chromatin into heterochromatin and euchromatin largely depends on pathways that engage DNA (i.e., replication and transcription). When damage occurs at regions of DNA that are being replicated or transcribed, the coordination of these events relies heavily on the multivalency of the regulatory factors involved. Interestingly, a growing body of evidence supports a model where ongoing transcription can promote DSB repair through homologous recombination (Ouyang et al., 2017). In a compact, heterochromatic environment, DNA repair is challenged by a high density of repetitive sequences and hindered access to the damaged DNA. The density of heterochromatin is dramatic enough that it was recently found to behave as a solid structure (Strickfaden et al., 2020). The accessibility of damaged DNA to repair factors can be enhanced by specific signals that regulate the transition between different chromatin states, with histone acetylation being a prime example due to its impact on chromatin folding, as well as DNA repair (Eberharter and Becker, 2002; Rodriguez and Miller, 2014; Kim et al., 2019; Chen et al., 2021). However, histone acetylation alone was found to not be sufficient to induce liquid-like properties in DNA. It is likely that in order to repair DSBs within regions of the genome that are difficult to access (e.g., heterochromatin, replicating and or transcribing DNA), repair factors will need to overcome these barriers to access the DNA lesion. Given the diverse nature of chromatin and the activities of the genome within not only the same cell but between different cell types, we speculate that the mechanisms utilized by these factors may differ depending on chromatin states and genome location/process in which the DNA damage occurs. Taken as a whole, the diverse interactions of DNA repair factors on chromatin that are highlighted here provide a framework for considering the complexity of repairing a lesion within a chromatinized and functioning genome. It is fascinating to consider the diverse nature of these interactions that drive repair within chromatin and consider future studies aimed at refining our view of the regulatory mechanisms that ensure proper engagement of these signals by the DDR and chromatin factors to govern the maintenance of genome integrity.

## Author Contributions

AS and KM wrote the manuscript with assistance from DL and DK. AS and KM constructed the figures. All authors reviewed and finalized the final version of the manuscript.

## Conflict of Interest

The authors declare that the research was conducted in the absence of any commercial or financial relationships that could be construed as a potential conflict of interest.

## Publisher’s Note

All claims expressed in this article are solely those of the authors and do not necessarily represent those of their affiliated organizations, or those of the publisher, the editors and the reviewers. Any product that may be evaluated in this article, or claim that may be made by its manufacturer, is not guaranteed or endorsed by the publisher.
